# Pembrolizumab in the treatment of non-small cell lung cancer—experiences from clinical practice

**DOI:** 10.3389/fmed.2025.1635626

**Published:** 2025-09-01

**Authors:** Lora Novakovic Lackovic, Milica Srecic Tomic, Marko Novakovic, Mirko Turic, Aleksandra Kajkut, Teodora Macinkovic, Mirko Stanetic, Ranko Glamocak

**Affiliations:** ^1^Faculty of Medicine, University of Banja Luka, Banja Luka, Bosnia and Herzegovina; ^2^Clinic for Pulmonary Diseases of the University Clinical Centre of the Republic of Srpska, Banja Luka, Bosnia and Herzegovina; ^3^Department of Internal Medicine, University Clinical Center of Banja Luka, Banja Luka, Bosnia and Herzegovina

**Keywords:** carcinoma, non-small-cell lung, antibodies, monoclonal, immune checkpoint inhibitors

## Abstract

**Background:**

Immune checkpoint inhibitors (ICIs) have become the standard first-line treatment for patients with advanced or metastatic non-small cell lung cancer (NSCLC) without targetable mutations. This study aimed to assess real-world outcomes of pembrolizumab monotherapy in patients with high PD-L1 expression (≥50%) and compare them with results from the KEYNOTE-024 clinical trial.

**Methods:**

This retrospective study included patients with advanced or metastatic NSCLC treated with pembrolizumab as first-line therapy at the Clinic for Pulmonary Diseases, University Clinical Center of Republika Srpska, between January 2018 and December 2022. Clinical and pathological data were collected from medical records and analyzed using descriptive and inferential statistical methods.

**Results:**

The cohort included 46 patients with a median age of 64 years; 56.5% were aged ≥65, 73.9% were male, 76% were smokers, and 72% had an ECOG performance status of 1. Adenocarcinoma (AC) and squamous cell carcinoma (SCC) were diagnosed in 50 and 46% of cases, respectively, while 70% had metastatic disease and 15% had brain metastases. The two-year objective response rate (ORR) was 72.2%, lower than the 85.7% reported in KEYNOTE-024, possibly due to differences in PD-L1 assay (SP263 vs. 22C3) and patient selection. Despite this, the median overall survival (OS) was 36 months—higher than in the trial. One-, two-, and three-year survival rates were 57.9, 53.5, and 42.8%, respectively.

**Conclusion:**

Our findings confirm the clinical benefit of pembrolizumab in a real-world setting, despite lower ORR compared to clinical trial data. However, the prognosis remains guarded due to the advanced stage and comorbidities of the population. Further investigation is warranted to optimize patient selection and treatment strategies.

## Introduction

Recent advances in immunology and biotechnology—including the development of monoclonal antibodies, recombinant DNA technology, lymphocyte culture, and gene transfer techniques—have revitalized interest of immunotherapy as an established modality in cancer treatment. These innovations have positioned immunotherapy as the fourth modality of cornerstone of oncologic care, alongside surgery, chemotherapy, and radiotherapy (1–4).

The principal strategy of cancer immunotherapy is to modulate the immune system to recognize and eliminate malignant cells. Immune checkpoint inhibitors (ICIs), particularly those targeting the PD-1/PD-L1 axis, have transformed the therapeutic landscape of non-small cell lung cancer (NSCLC) Programmed death ligand 1 (PD-L1) is a transmembrane protein expressed on tumor cells and infiltrating immune cells. Its interaction with the PD-1 on activated T cells leads to immune suppression and facilitates tumor immune evasion (5).

PD-L1 expression is both a predictive and prognostic biomarker in NSCLC. As a predictive biomarker, PD-L1 helps identify patients likely to benefit from ICIs. High PD-L1 expression [Tumor Proportion Score (TPS) ≥ 50%] is associated with greater benefit from pembrolizumab monotherapy, while patients with lower expression (TPS 1–49%) may benefit more often treated with combination regimens. Those with negative PD-L1 expression (TPS < 1%) are generally not candidates for monotherapy but may respond to chemo-immunotherapy combinations.

PD-L1 is commonly assessed by immunohistochemistry (IHC), and scoring systems include the TPS for tumor cells and Immune Cell (IC) score, mainly used with atezolizumab. However, PD-L1 assessment has limitations, including inter- and intra-tumoral heterogeneity, temporal variability, and differences in staining protocols. Variability across IHC assays—such as 22C3, 28-8, SP263, and SP142—adds further complexity, as shown in Table 1, with each assay being linked to a specific therapeutic agent and platform (6–8).

**Table 1:** Commercially available PD-L1 IHC tests.

IHC test	Antibody	Platform	Applied PD-1/PD-L1 inhibitor	Target cells
22C3 pharmDx	Monoclonal antibody 22C3	Dako	Pembrolizumab	Tumor cells (TPS)
28-8 pharmDx	Monoclonal antibody 28-8	Dako	Nivolumab	Tumor cells (TPS)
SP263*	Monoclonal antibody SP263	Ventana	Durvalumab	Tumor cells (TPS)
SP142	Monoclonal antibody SP142	Ventana	Atezolizumab	Immune cells (IC) and tumor cells (TPS)

*PD-L1 testing was conducted with the SP263 assay, which is our institution’s standard practice based on platform availability and cost considerations. While 22C3 is the FDA-approved companion diagnostic assay for pembrolizumab, SP263 has shown equivalent analytical performance in several studies (13–15). Nevertheless, some studies have reported systematic differences between the assays, with SP263 potentially yielding higher PD-L1 scores and leading to patient reclassification (16). SP263 is therefore routinely utilized in real-world practice when 22C3 is not accessible. This approach is consistent with routine clinical practice in many institutions throughout Europe, where assay selection is often influenced by local laboratory infrastructure and resource constraints (6–8).

The use of ICIs has significantly improved clinical outcomes for NSCLC patients, particularly those with high PD-L1 expression—reported in approximately 23–28% of advanced cases (9).

Pembrolizumab is a highly selective humanized monoclonal antibody that blocks the PD-1 receptor, preventing its interaction with PD-L1 and PD-L2. Based on findings from pivotal trials, including KEYNOTE-024, pembrolizumab was approved as first-line monotherapy for advanced and/or metastatic NSCLC with high PD-L1 expression. KEYNOTE-024 demonstrated that pembrolizumab significantly improved both overall survival (OS) and progression free survival (PSF) compared with platinum based chemotherapy (9). Long-term data confirmed this benefit, reporting a five-year OS rate of 31.9% in pembrolizumab versus 16.3% with chemotherapy (10). These findings have led to changes in global therapeutic guidelines and the introduction of pembrolizumab as the standard first-line treatment for NSCLC.

This study aims to evaluate real-world outcomes of pembrolizumab monotherapy in patients with advanced and metastatic NSCLC and high PD-L1 expression, with a particular focus on overall survival and treatment response compared to results from the KEYNOTE-024 trial.

### Aim of the study

The aim of the present research is to evaluate the result of first-line pembrolizumab monotherapy in metastatic NSCLC patients with high PD-L1 expression.

Despite robust trial data, real-world outcomes may vary due to population differences, alternative assays, or clinical setting limitations.

## Materials and methods

A retrospective/prospective study conducted at the Clinic for Pulmonary Diseases University Clinical Center included patients in the period January 1, 2018 and December 31, 2022. The prospective part of the research lasted until the deadline for the follow-up of the respondents and was completed on December 31, 2024.

The study protocol was reviewed and approved by the Ethics Committee of the University Clinical Center of the Republic of Srpska (Approval number: 01-19-115/25). Given the retrospective design and the use of de-identified clinical data, informed consent was waived. All procedures were conducted in accordance with institutional guidelines and the Declaration of Helsinki.

### Patients

The study included 46 patients with histologically confirmed NSCLC, diagnosed by the Institute of Pathology at the University Clinical Center of Republika Srpska. Eligible patients had high PD-L1 expression (TPS ≥ 50%) as determined by the SP263 assay and tested negative for activating EGFR mutations and ALK rearrangements (wild-type). All patients were treatment-naive in the advanced or metastatic setting and received pembrolizumab monotherapy as first-line systemic therapy, in accordance with international guidelines (e.g., NCCN, ESMO). Patients with actionable driver mutations, prior systemic treatment for advanced disease, ECOG performance status >2, or contraindications to immunotherapy were excluded.

### Study design

Enrolled patients were chemo-naive or had previously undergone palliative radiotherapy. All patients received a fixed dose of 200 mg pembrolizumab administered every 3 weeks. Treatment response was measured by applying RECIST version 1.1 after a treatment duration of 2 years. RECIST 1.1 response assessment was determined retrospectively by one experienced medical oncologist using available imaging records. Centralized or double-reader imaging review could not be performed due to the retrospective real-world study design. Hence, inter-rater variability could not be officially determined.

The evaluator was blinded to clinical outcomes to minimize potential bias, however. Clinical characteristics and all the treatment-related information were obtained from medical history.

PD-L1 expression was assessed using the SP263 antibody on the Ventana BenchMark platform, which is routinely used at our institution. This choice reflects an institutional preference primarily driven by the availability of the platform and cost considerations, rather than any scientific or clinical superiority of one assay over another. While the SP263 assay is not the FDA-approved companion diagnostic for pembrolizumab, it is widely used in clinical practice and has shown comparable performance to the 22C3 assay in several studies. We confirm that there are no vendor relationships or financial incentives influencing this choice.

The stage of disease was classified according to the eighth edition of the TNM staging for NSCLC.

### Statistical analysis

Statistical analysis was performed using SPSS for Windows (Version 20; SPSS, Chicago, IL, United States). Descriptive data for all groups and variables will be presented as numbers and percentages, and compared with the *χ*^2^ test. The independent variable used in the research is a therapeutic response. A statistically significant difference was defined at the *p* < 0.05 level and a difference of very high statistical significance at the *p* < 0.01 level.

## Results

A total of 46 patients with advanced or metastatic NSCLC were included in the study. All patients had high PD-L1 expression (Tumor Proportion Score ≥50%), and tested negative for activating mutations, such as EGFR mutations or ALK rearrangements. Pembrolizumab was administered as first-line monotherapy, with planned treatment duration of up to 2 years.

By the end of the two-year period, 21 patients had died, while 25 remained alive and were available for analysis of treatment duration and outcomes beyond the initial 2 years.

The median age at diagnosis was 64 years (range: 37–78), with 56.5% of patients aged ≥65 years. The majority were male (73.9%) and current or former smokers (76%). In terms of histological subtypes, 50% of patients had adenocarcinoma (AC), 46% had squamous cell carcinoma (SCC), and 4% had NSCLC not otherwise specified (NOS).

Most patients (70%) presented with metastatic (stage IV) disease, and 15% had brain metastases at the time of diagnosis. Regarding functional status, 72% of patients had an ECOG performance status of 1, and 28% had ECOG 0 at baseline.

These baseline demographic and clinical characteristics are summarized in Table 2.

**Table 2:** Baseline demographic and clinical characteristics of the study population (*N* = 46).

Variable	*n* (%)		*n* (%)
Age at diagnosis (median 64 years)	<65		≥65
	20 (43.5%)		26 (56.5%)
Gender	Male		Female
	34 (73,9%)		12 (26,1%)
Smoking status	Smoker		Non-smoker
	35 (76%)		11 (24%)
Histology	Squamous	Adeno	NSCLC NOS
	21 (46%)	23 (50%)	2 (4%)
Stage of the disease	IIIB and IIIC		IVA and IVB
	14 (30%)		32 (70%)
ECOG PS	0		1
	13 (28%)		33 (72%)
Brain metastases	Yes		No
	7 (15%)		39 (85%)

Data are presented as n (%) unless otherwise specified. NSCLC NOS: non-small cell lung cancer not otherwise specified; ECOG PS, Eastern Cooperative Oncology Group performance status.

The objective response rate (ORR) after 2 years of treatment is 72.2%. The one-year survival rate is 57.9%, the two-year survival rate is 53.5%, and the three-year survival rate is 42.8%.

**Figure 1:**
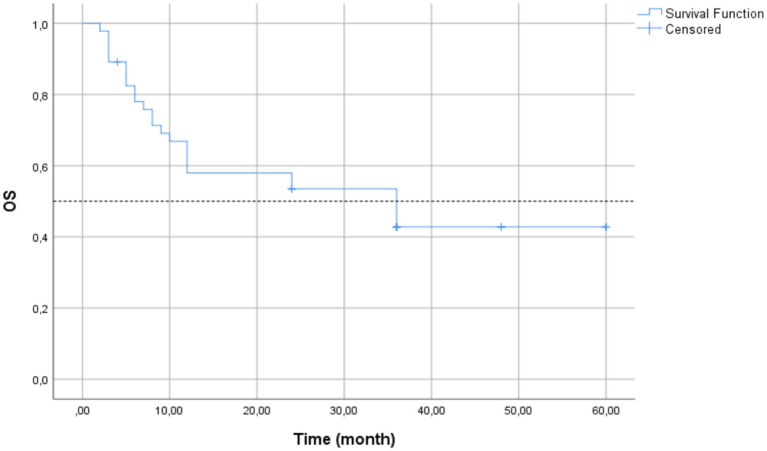
Kaplan–Meier curve of overall survival (OS) in patients with advanced NSCLC and high PD-L1 expression treated with first-line pembrolizumab—the solid line represents the OS probability over time, showing an initial decline (early mortality subset) followed by a plateau (longer survivors). The median OS was 36.0 months (95% CI: 12.3–59.7), and the mean OS was 33.5 months (95% CI: 26.3–40.8). Censored observations marked by + symbols, represent patients alive at last follow-up or lost to follow-up. The dotted line represents the 50% survival threshold.

The Kaplan–Meier curve for overall survival (OS) is presented in Figure 1. The curve demonstrates a steep initial decline during the first 10 months of follow-up, indicating early mortality among a subset of patients.

This is followed by a plateau phase, reflecting improved survival among the remaining patients. The mean overall survival time was 33.50 months (95% CI, 26.25–40.75), with a standard error of 3.70 months. The median overall survival was 36.00 months (95% CI, 12.31–59.69), with a standard error of 12.09. Estimation of median survival was limited to the largest observed survival time, which was censored.

The multivariable Cox proportional hazard model was employed to assess the association between gender and risk of adverse events, with women as the reference group. The analysis revealed that men had HR = 0.465 (95% CI, 0.159–1.361, *p* = 0.162), suggesting a non-statistically significant trend toward a lower risk of adverse events compared to women.

The mean overall survival time for the entire cohort was 33.50 months (95% CI: 26.25–40.75). The median survival time was 36 months (95% CI: 12.31–59.69).

**Figure 2:**
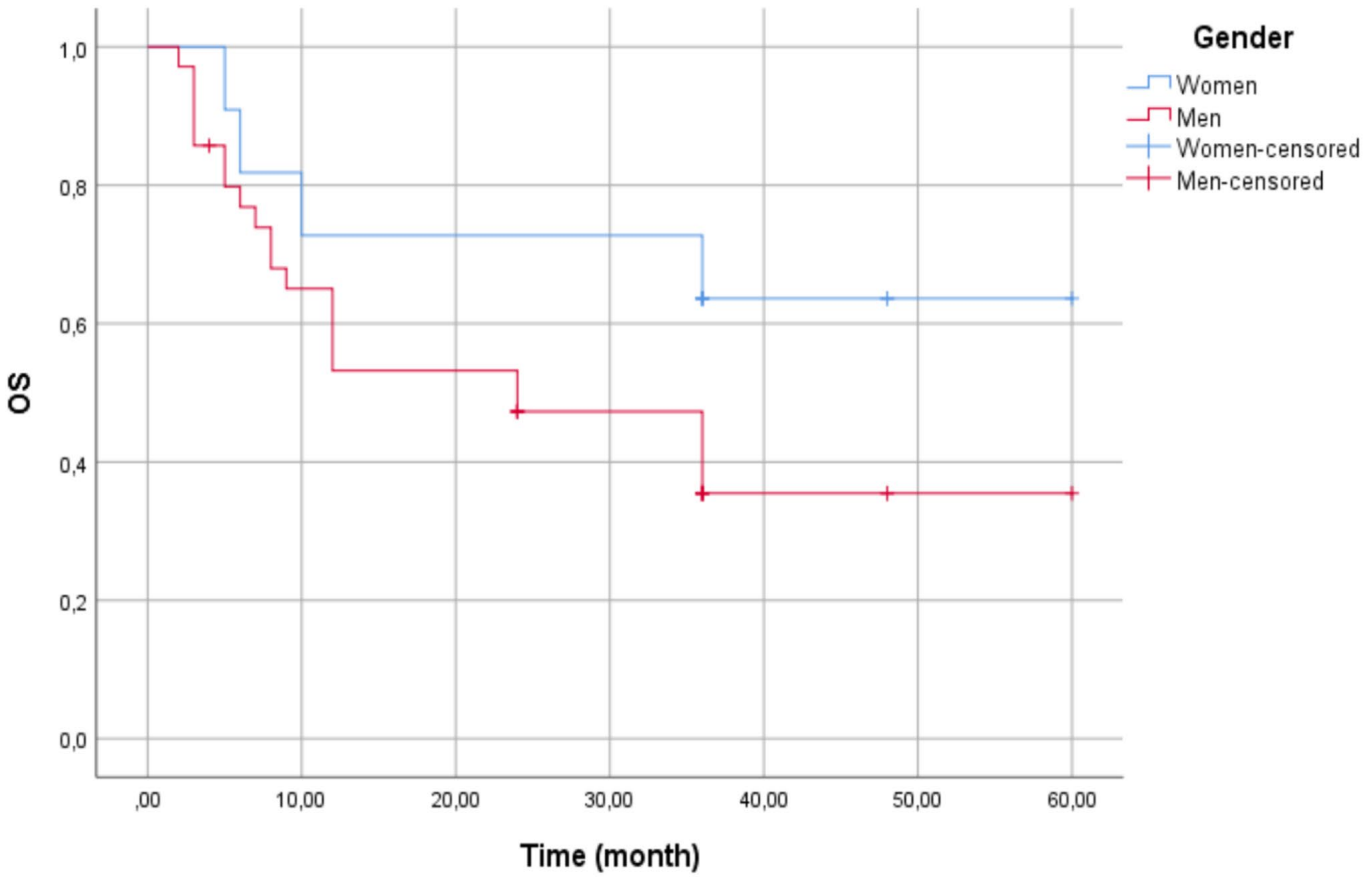
Kaplan–Meier survival curves by gender—the overall mean survival time was 33.50 months (95% CI: 26.25–40.75). Women (blue line) showed a longer mean survival (43.36 months, 95% CI: 29.59–57.13) compared to men (red line; 30.19 months, 95% CI: 21.98–38.40). Median survival was 24 months for men (95% CI: 8.67–39.33) but could not be estimated for women due to high censoring (75% censored). The Log-Rank test indicated no statistically significant difference between groups (*χ*^2^(1) = 2.202, *p* = 0.138). Censored observations are marked with vertical ticks.

The Kaplan–Meier survival estimate revealed that females exhibited a longer mean survival time of 43.36 months (95% CI: 29.59–57.13; SE: 7.03 months). However, the median survival time could not be computed due to high censoring (75% of female patients were still alive at data cutoff), limiting event observations. Men had a shorter mean survival time of 30.19 months (95% CI: 21.98–38.40; SE: 4.19 months) and a median survival of 24 months (95% CI: 8.67–39.33; SE: 7.82 months) (Figure 2).

The Kaplan–Meier survival curves were compared using the Log-Rank test (Mantel-Cox), yielding *χ*^2^(1) = 2.202 (*p* = 0.138), excluding statistically significant survival distributions between gender at the 0.05 level.

The multivariable Cox regression analysis showed no significant association between age group (<65 vs. ≥65 years) and event hazard (HR = 1.247, 95% CI: 0.57–2.74; *p* = 0.581). The overall cohort mean survival time for all patients was 33.50 months (95% CI: 26.25–40.75), and median survival was 36.00 months (95% CI: 25.41–46.59).

The mean survival time for patients aged below 65 years was recorded as 27.87 months (95% CI: 20.20–35.53) with a standard error of 3.91 months. Median survival was 36.00 months (95% CI: 25.41–46.59) with a standard error of 5.40 months.

**Figure 3:**
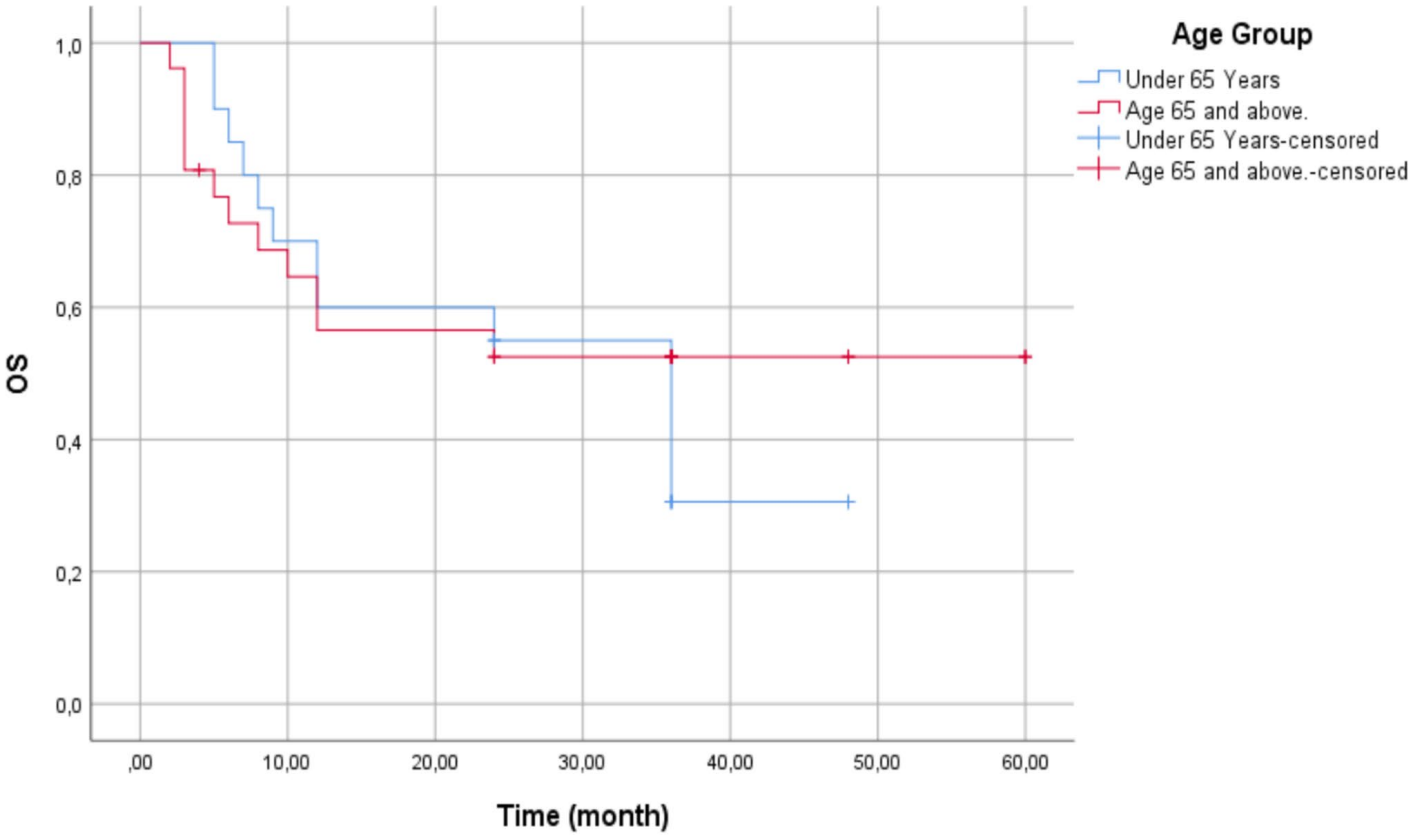
Kaplan–Meier survival curves by age group (<65 vs. ≥65 years)—patients aged ≥65 years (blue line) showed a non-significantly longer mean survival (35.15 months) compared to those <65 years (red line; 27.87 months). Median survival was 36.00 months for the <65 group but inestimable for ≥65 due to censoring (vertical ticks). The Log-Rank test confirmed no statistically significant difference (*p* = 0.567).

However, the patients aged 65 years and older had a longer mean survival time of 35.15 months (95% CI: 24.82–45.48) with a standard error of 5.27 months (Figure 3). The median survival time for patients aged ≥65 years could not be calculated due to censoring, indicating a substantial proportion of these patients may have survived beyond the study period.

Log-Rank test (Chi-Square) gives a *χ*^2^(1) = 0.327 and *p*-value = 0.567, excluding statistically significant survival distributions between the two age groups at the 0.05 level.

The multivariable Cox regression revealed no statistically significant association between smoking status and survival outcomes (HR = 0.852, CI: 0.541–1.342, *p* = 0.490). Compared to current smokers (reference group), other smoking categories (non-smokers, former smoking, unknown status) showed a non-significant 15% reduction in hazard risk.

**Figure 4:**
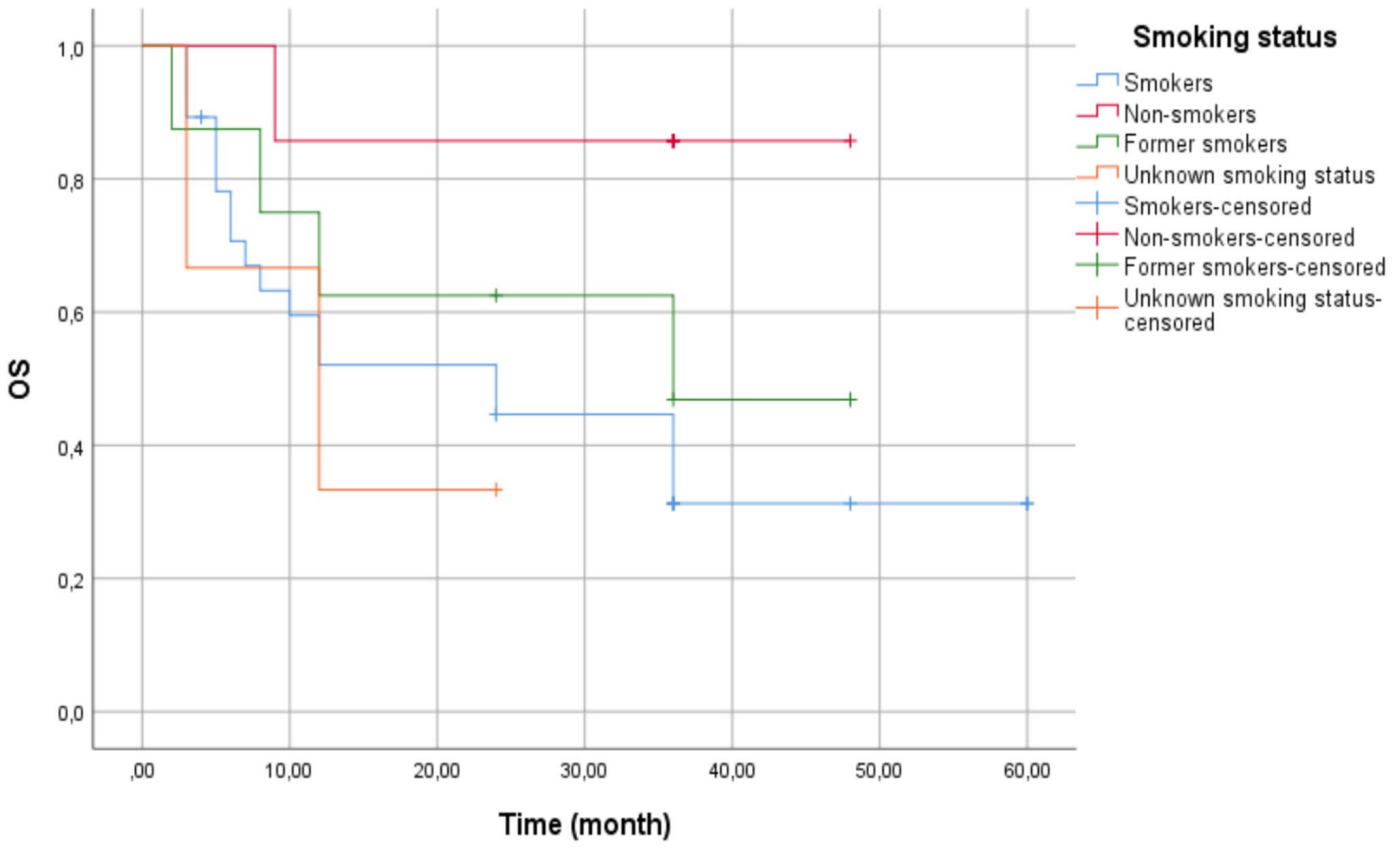
Kaplan–Meier survival curves by smoking status—current smokers (blue line) showed the shortest mean survival (28.51 months). Non-smokers (red line) demonstrated the longest mean survival (42.43 months), though median survival was inestimable due to censoring. Former smokers (green line) and unknown status (orange line) showed intermediate outcomes. Vertical ticks indicate censored cases. Log-Rank test revealed no statistically significant differences between groups (*p* = 0.132).

The overall cohort mean survival was 33.50 months (95% CI: 26.25–40.75) (Figure 4). Current smokers had a mean survival time of 28.51 months (95% CI: 19.55–37.47) with a standard error of 4.57 months. Non-smokers exhibited the highest mean survival time of 42.43 months (95% CI: 32.32–52.54) with a standard error of 5.16 months. Former smokers had a mean survival time of 30.88 months (95% CI: 17.73–44.02) with a standard error of 6.71 months. Patients with unknown smoking status displayed the lowest mean survival time at 13.000 months (95% CI: 3.27–22.73) with a standard error of 4.97 months.

The median survival time for current smokers was 24.00 months (95% CI: 6.35–41.65) with a standard error of 9.00 months. The median survival time for former smokers was 36.00 months, but confidence intervals could not be computed due to censoring. Patients with unknown smoking status had a median survival time of 12.00 months (95% CI: 0.00–26.40) with a standard error of 7.35 months. The median survival time for non-smokers was not estimable, potentially due to a high proportion of censored cases.

The Log-Rank test (Mantel-Cox) showed no significant difference in survival distributions across groups (*χ*^2^(3)= 5.607, *p* = 0.132).

The Cox regression showed no significant survival difference by histology (AC reference) HR = 1.107, 95% CI: 0.56–2.19, *p* = 0.769. The Wald statistic (0.086) reports this lack of evidence for a tumor type effect on the hazard.

**Figure 5:**
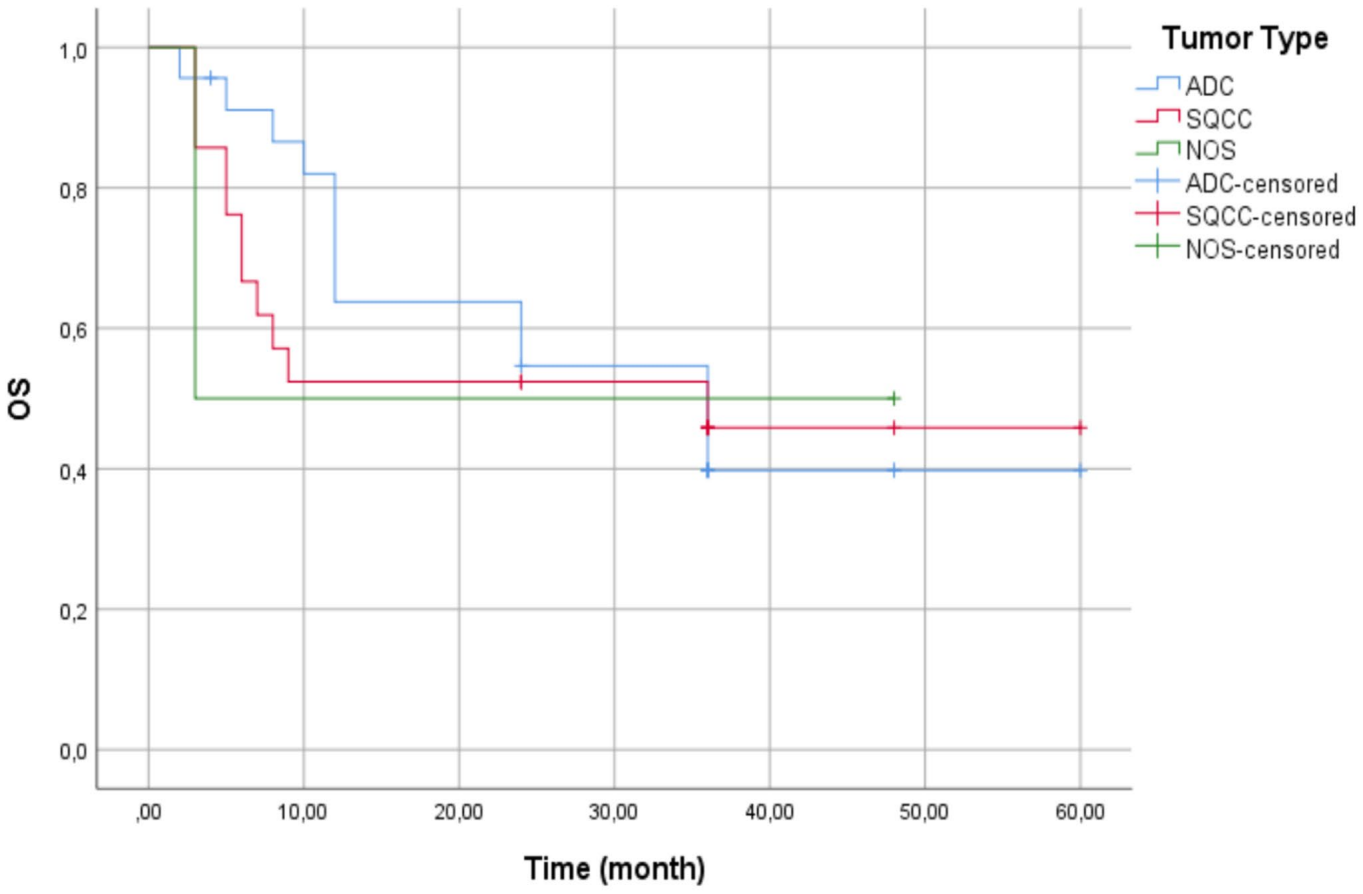
Kaplan–Meier survival curves by histologic subtype—patients with adenocarcinoma (blue line) showed the longest mean survival (34.73 months). Those with squamous cell carcinoma (red line) demonstrated intermediate outcomes (32.48 months), and patients with non-small cell lung cancer not otherwise specified (green line) had the poorest survival (25.50 months). Vertical ticks indicate censored cases. No statistically significant differences between groups (Log-Rank *p* = 0.953).

The overall cohort mean survival was 33.50 months (95% CI: 26.25–40.75). Patients with AC tumors exhibited the highest mean survival time at 34.73 months (95% CI: 25.28–44.17) with a standard error of 4.82 months. Patients with SCC tumors had a mean survival time of 32.48 months (95% CI: 21.09–43.86) with a standard error of 5.81 months. Patients with NCSLC NOS tumors had the lowest mean survival time at 25.50 months (95% CI: 0.00–56.68) with a standard error of 15.91 months (Figure 5).

The overall cohort median survival was 36.00 months (95% CI: 12.31–59.69), consistent with the median for AC and SCC tumor patients. For patients with AC tumors, the median survival time was 36.00 months (95% CI: 15.14–56.86) with a standard error of 10.64 months.

For patients with SCC tumors, the median survival time was 36.00 months, but confidence intervals could not be calculated due to censoring. For patients with NSCLC NOS tumors, the median survival time was 3.00 months, reflecting substantially shorter survival compared to the other groups.

The Log-Rank test showed no significant survival difference between groups (*χ*^2^(2)= 0.097, *p* = 0.953). The NSCLC-NOS category included only tumors that remained unclassifiable after standard diagnostic procedures (exclude rare subtypes like sarcomatoid carcinoma).

While a comprehensive analysis of immune-related adverse events (irAEs) was beyond the scope of this study, the most commonly observed irAEs included endocrinopathies such as hypo- or hyperthyroidism, dermatologic manifestations such as pruritus, and occasional cases of colitis. These events were generally managed symptomatically or with corticosteroids and, in the majority of cases, did not require permanent pembrolizumab discontinuation.

However, detailed data on the frequency and severity of irAEs were not systematically collected in this cohort. These observations are in line with has been previously published regarding pembrolizumab safety profiles in NSCLC patients, where on thyroid dysfunction, skin toxicity, and gastrointestinal events represent some of the most common irAEs reported (11, 12).

## Discussion

The findings of this research identify several clinically relevant outcomes from the use of pembrolizumab as first-line therapy in patients with NSCLC with high PD-L1 expression.

PD-L1 testing in our cohort was performed using the SP263 assay, which, according to some studies, may yield higher PD-L1 scores and potentially lead to patient misclassification and inappropriate selection for monotherapy. While the FDA has approved pembrolizumab specifically with the 22C3 assay as a companion diagnostic, the European Medicines Agency (EMA) allows for greater flexibility, accepting other analytically validated PD-L1 assays in routine clinical practice. This regulatory variability may have contributed to differences in PD-L1 classification and treatment outcomes observed in our cohort. These findings underscore the importance of careful interpretation when alternative assays are used and support ongoing efforts to harmonize PD-L1 testing in NSCLC immunotherapy. The use of SP263 as a substitute for 22C3 may be acceptable under certain clinical circumstances, but the analytical and clinical concordance must be carefully considered. Multiple studies have evaluated the concordance between 22C3 and SP263 assays. In the IMpower010 trial, Zhou et al. (13) reported a high concordance rate of 91.8% at the ≥50% PD-L1 expression cutoff. Similarly, Kim et al. (14) and Fujimoto et al. (15) demonstrated overall agreement rates of 94.5 and 92.6%, respectively, at the high-expression threshold.

However, other studies have highlighted critical discrepancies, especially at clinically actionable cutoffs (≥1% and ≥50%). Munari et al. (16) reported that SP263 frequently yielded higher PD-L1 scores compared to 22C3, which resulted in patient reclassification and potentially altered therapeutic decisions, especially around eligibility for pembrolizumab monotherapy. Beyond assay variability, PD-L1 expression itself is known to be both spatially and temporally heterogeneous. Intra-tumoral heterogeneity may lead to sampling bias, as PD-L1 levels can differ across various tumor regions or between primary and metastatic sites. Small or single-site biopsies may thus fail to capture the full PD-L1 profile of the tumor (17–19).

Additionally, PD-L1 expression may change over time, particularly following chemotherapy, radiotherapy, or targeted therapies. Such temporal variability could result in discordant findings between initial diagnostic and later-stage samples (5, 20). In our study, PD-L1 testing was performed at baseline using treatment-naive samples, which strengthens internal consistency, but the absence of longitudinal assessment is a recognized limitation. Future studies should aim to include serial or multi-site sampling to better characterize PD-L1 dynamics and optimize treatment selection.

An extensive analysis was conducted on 46 patients, 21 of whom died during the two-year follow-up period. The remaining 25 patients were available for continued evaluation beyond the planned two-year treatment duration. Among these, patients who had achieved complete response (CR), partial response (PR), or stable disease (SD) were managed through routine clinical follow-up without further systemic therapy. In contrast, those who experienced progressive disease (PD) were transitioned to alternative treatments, including second-line chemotherapy or palliative radiotherapy, depending on clinical indications and performance status. This post-treatment management approach provides additional insight into the durability and limitations of pembrolizumab monotherapy in real-world settings.

The median age shows that the sample was more or less equally distributed among younger and older participants. The patterns of lung cancer patient ages have also changed clinically and epidemiologically. Over the past decades, a notable trend for declining average ages at diagnosis has been observed. While the median age at diagnosis is 71 years, around 6.0% of diagnoses occur in individuals younger than 55 years. Moreover, diagnoses of lung cancer in those younger than 45 years of age and 35 years of age remain infrequent, accounting for around 1.1 and 0.2% of all cases, respectively (21–23). The majority of participants are male (73.9%), consistent with known data showing a higher prevalence of lung cancer among men, especially in smoking populations. A high percentage of participants (76%) are smokers, reinforcing the strong link between smoking and lung cancer.

Evidence from conducted studies suggests that pembrolizumab as a monotherapy or in combination with chemotherapy for 2 years is able to yield high rates of objective response (ORR) and long survival in patients with NSCLC. In a study, an objective response rate of 85.7% was obtained (24). In second study, following an interval of 3 years, patients treated for 2 years had an objective response rate of 82% (25).

The lower ORR observed in our study (72.2%) compared to pivotal clinical trials such as KEYNOTE-024 (85.7%) may be attributed to the use of a different PD-L1 assay (SP263 antibody-based). This methodological variation could lead to an overestimation of PD-L1 expression, potentially resulting in a reduced therapeutic response. Additionally, the use of archival tissue samples may compromise biomarker stability and accuracy, contributing to potential misclassification of PD-L1 status. A methodological limitation of our study is the utilization of archival tissue samples for PD-L1 testing. While this strategy mimics standard clinical practice—where re-biopsy is not typically done before starting immunotherapy—it is a concern that biomarkers may have degraded over time. That said, previous studies indicate that PD-L1 expression in FFPE tissue is mostly unchanged when samples are well fixed and stored under controlled conditions.

In our cohort, all samples were processed according to standard institutional procedures, and the duration from biopsy to testing was within a window that is generally acceptable for real-world biomarker studies. Nevertheless, the risk of PD-L1 misclassification from extended storage cannot be fully discounted and needs to be taken into consideration when interpreting our findings (26–28). However, our findings are consistent with other real-world studies using the SP263 assay. For instance, Fujimoto et al. (29) reported an ORR of 70.2% in patients with high PD-L1 expression (≥50%) assessed by SP263, which aligns closely with our results. This suggests that the observed difference in ORR may not solely represent methodological limitations, but rather reflects assay performance and the heterogeneity of patients typically seen in clinical practice.

Furthermore, inconsistencies in the application of RECIST criteria across evaluators may have impacted the assessment of objective response. In our study, RECIST version 1.1 was applied retrospectively by a single experienced oncologist, based on available imaging. Due to the retrospective nature of the data and lack of infrastructure for centralized radiologic review, inter-observer agreement could not be quantified. This represents a methodological limitation; however, the evaluator was blinded to clinical outcomes in an effort to reduce bias.

When interpreting survival outcomes, it is important to consider the potential influence of survivorship bias. Patients who remained on pembrolizumab for extended periods and were alive at the end of follow-up may represent a biologically favorable subgroup with inherently better prognosis, potentially inflating survival estimates.

Additionally, due to the high rate of censored observations—particularly among female and elderly patients—median survival for some subgroups could not be calculated, further limiting interpretability. This pattern of right-censoring introduces uncertainty regarding the true long-term survival and may disproportionately reflect patients who tolerate therapy well. Consequently, these results should be interpreted with caution and not generalized to all patients with advanced NSCLC and high PD-L1 expression.

In the phase II randomized trial KEYNOTE-024, pembrolizumab exhibited a median overall survival (OS) of 30 months in patients with high PD-L1 expression, significantly greater than the chemotherapy arm with a median OS of 14.2 months (30). In an observational multicenter study, the median OS was found to be 19.1 months with a five-year survival rate of 24.8% (31).

The outcomes of our investigation present a median OS of 36 months, higher than what was reported in the studies outlined above. This apparent survival advantage may reflect differences in baseline clinical characteristics, particularly patient selection. Our cohort had a lower proportion of patients with brain metastases (10.8%) at diagnosis, and most patients received immunotherapy as first-line treatment with high PD-L1 expression confirmed prior to therapy. Furthermore, patients who survived long enough to complete the two-year treatment course may represent a more immunoresponsive subgroup. These factors may have contributed to the prolonged OS observed in our analysis.

The Kaplan–Meier survival analysis revealed that the female patients had a longer mean survival time in comparison to the male patients (43.36 months vs. 30.19 months), but the difference was not statistically significant. The same trends have been observed in previous studies, where sex was not found to be an independent overall survival prognostic factor (32). The absence of statistical significance in the present study may be due to the small sample size.

Besides, it was observed that elderly patients (≥65 years) had higher mean survival compared to younger patients (<65 years) (35.15 vs. 27.87 months). As the observed difference did not achieve statistical significance, the meaning of these results is the age is not an independent prognostic variable and that treatment success is similar in both age groups. Our findings are in agreement with the literature available, and as can be seen, age does not seem to have any role in OS for PD-1/PD-L1 inhibitor-treated NSCLC patients (33, 34).

While non-smokers in our study had the longest average overall survival (42.43 months), this was not statistically significant and could easily not be representative of true biological distinctions. Interestingly, this is contrary to the current literature because nowadays research shows, that non-smokers often show a lesser response to immunotherapy in NSCLC than smokers and ex-smokers. The enhanced response to immune checkpoint inhibitors observed in former smokers, as compared to never-smokers, is believed to result from sustained molecular damage and host-related factors that resemble those found in current smokers—including a high tumor mutational burden (TMB) and an inflamed tumor microenvironment. Smoking induces a range of molecular alterations in tumor cells, contributing to an increased tumor mutational burden (TMB). A higher TMB is associated with enhanced neoantigen generation, which may improve tumor immunogenicity and response to immunotherapy (35–37).

Never-smokers, on the other hand, often have oncogenic driver mutations like EGFR or ALK, which are linked to lower TMB and less benefit from PD-1/PD-L1 inhibition (38). TMB data were not available in our study, making us unable to correlate molecular characteristics with clinical outcomes. The improved survival in non-smokers is hence more likely due to other confounding variables, including more favorable baseline clinical features or statistical fluctuation from the relatively small sample size. The findings should therefore be interpreted cautiously and not as proof of better immunotherapy effectiveness in never-smokers.

The longest mean survival was observed in patients with adenocarcinoma (34.73 months), while the shortest was recorded in patients with NSCLC NOS (25.5 months). As the differences observed were not statistically significant, the analysis did not establish histological subtype as a significant prognostic factor (31). According to the literature, patients with squamous cell carcinoma of the lung often demonstrate a better therapeutic response to immunotherapy, which is attributed to the biological and immunological characteristics of this histological subtype. Squamous cell carcinoma is strongly associated with smoking, resulting in a higher tumor mutational burden (TMB) and more frequent high PD-L1 expression, both of which increase the likelihood of response to immune checkpoint inhibitors (ICI). In a study where patients with squamous cell lung carcinoma treated with nivolumab in the second-line setting showed significantly longer OS and better response rates compared to chemotherapy, these findings provided key evidence leading to the widespread adoption of immunotherapy in squamous cell lung carcinoma (39).

Although results have shown that ICIs can improve both OS and progression-free survival (PFS) in patients with both squamous and non-squamous lung cancer, a significantly greater survival benefit from ICIs was observed among those with squamous cell carcinoma (40).

## Conclusion

Although survival outcomes varied by sex, age, histology, and smoking status, none of the differences were statistically significant, likely due to limited statistical power. For example, in the comparison between smokers (*n* = 38) and non-smokers (*n* = 8), a post-hoc power analysis indicated approximately 25–30% power to detect a hazard ratio of 0.5 at *α* = 0.05. These limitations underscore the need for larger studies to confirm potential subgroup effects. Differences in outcomes compared to clinical trials may also reflect the use of the SP263 assay instead of 22C3, the relatively small and heterogeneous real-world population, and the retrospective single-center design.

These factors limit generalizability and may contribute to response variability. Despite these limitations, the study provides valuable insight into real-world application of pembrolizumab monotherapy in patients with high PD-L1 expression and no actionable mutations. Future studies should directly compare different PD-L1 assays, incorporate complementary biomarkers (e.g., TMB, TILs), and evaluate alternative therapeutic strategies, including combination approaches with chemotherapy, radiotherapy, or other immunomodulatory agents. Prospective, multicenter, and adequately powered trials are essential to better define treatment predictors and optimize immunotherapy personalization for advanced NSCLC.

## Data Availability

The datasets presented in this study can be found in online repositories. The names of the repository/repositories and accession number(s) can be found in the article/supplementary material.
